# ‘I stayed with my illness’: a grounded theory study of health seeking behaviour and treatment pathways of patients with obstetric fistula in Kenya

**DOI:** 10.1186/s12905-017-0451-6

**Published:** 2017-09-29

**Authors:** Anne M. Khisa, Grace M. Omoni, Isaac K. Nyamongo, Rachel F. Spitzer

**Affiliations:** 1 University of Nairobi, School of Nursing Sciences, Box 11952-00100, Nairobi, PO Kenya; 2 University of Nairobi, School of Nursing Sciences, P.O Box 30197-00100, Nairobi, Kenya; 3 University of Nairobi, Institute of Anthropology, Gender and African Studies, P.O. Box 30197-00100, Nairobi, Kenya; 4 The Cooperative University of Kenya, Division of Cooperative Development, Research and Innovation, Box 24814-00502, Nairobi, PO Kenya; 5 University of Toronto, Dalla Lana School of Public Health, 155 College St. ON M57 3M7, Toronto, Canada

**Keywords:** Obstetric fistula, Health seeking behaviour, Grounded theory, Narratives, Kenya

## Abstract

**Background:**

Obstetric fistula classic symptoms of faecal and urinary incontinence cause women to live with social stigma, isolation, psychological trauma and lose their source of livelihoods. There is a paucity of studies on the health seeking behaviour trajectories of women with fistula illness although women live with the illness for decades before surgery. We set out to establish the complete picture of women’s health seeking behaviour using qualitative research. We sought to answer the question: what patterns of health seeking do women with obstetric fistula display in their quest for healing?

**Methods:**

We used grounded theory methodology to analyse data from narratives of women during inpatient stay after fistula surgery in 3 hospitals in Kenya. Emergent themes contributed to generation of substantive theory and a conceptual framework on the health seeking behaviour of fistula patients.

**Results:**

We recruited 121 participants aged 17 to 62 years whose treatment pathways are presented. Participants delayed health seeking, living with fistula illness after their first encounter with unresponsive hospitals. The health seeking trajectory is characterized by long episodes of staying home with illness for decades and consulting multiple actors. Staying with fistula illness entailed health seeking through seven key actions of staying home, trying home remedies, consulting with private health care providers, Non-Governmental organisations, prayer, traditional medicine and formal hospitals and clinics. Long treatment trajectories at hospital resulted from multiple hospital visits and surgeries. Seeking treatment at hospital is the most popular step for most women after recognizing fistula symptoms.

**Conclusions:**

We conclude that the formal health system is not responsive to women’s needs during fistula illness. Women suffer an illness with a chronic trajectory and seek alternative forms of care that are not ideally placed to treat fistula illness. The results suggest that a robust health system be provided with expertise and facilities to treat obstetric fistula to shorten women’s treatment pathways.

**Electronic supplementary material:**

The online version of this article (10.1186/s12905-017-0451-6) contains supplementary material, which is available to authorized users.

## Background

Obstetric fistula illness is a type of vaginal fistula caused by prolonged obstructed labour where the vaginal cavity communicates with the bladder or rectum [1] and is the most common form of vaginal fistula in resource limited countries [2]. The symptoms of vaginal fistula include urine incontinence, faecal incontinence, foot drop, infertility, skin ulceration [2, 3] and depression [4, 5]. Often, the illness classic symptoms of faecal and urinary incontinence cause the participants to live socially isolated lives and psychological trauma besides losing their daily source of livelihoods.

Existing research has focused on the surgical approaches in the management of fistula [6–8] the ethical issues surrounding care of fistula patients [9, 10] the characteristics of fistula patients that contribute to fistula formation [11–14] and the experiences of patients after fistula surgery and outcomes of treatment [15, 16]. Mselle and Kohi argue that sociocultural perceptions and practices of people on women who live with fistula affect women’s experiences of the illness [17]. However, there is a paucity of studies on the health seeking behaviour trajectories of women with obstetric fistula illness. Studies have indicated that women live with the illness for many years before surgery [4, 18]. However, the individual and contextual factors that surround and influence fistula patients’ health seeking behaviour and determine their treatment pathways remain unexplored.

Obstetric fistula is a devastating illness that affects 1% (or a quarter million) of women in Kenya [19]. The illness is treatable by corrective surgery, with an estimated 1200 women undergoing surgery annually [20]. The number of surgical operations is however insufficient given the large estimated number of women with fistula in the country. Efforts to treat women with fistula in Kenya have increased in the last decade. Surgeons have teamed up in select national referral and teaching hospitals and together with Nongovernmental organizations and offered free surgery to women suffering from obstetric fistula. There remain ongoing efforts to increase access to surgery for those who require it and to increase awareness creation in the public about fistula illness.

Kenyan fistula patients spend a long time, even decades before accessing surgery [4, 18]. However, there exists little information on how fistula patients navigate the treatment choices available to them and the type of therapy accessed by fistula patients remains unexplored. Further, little information is sought in retrospect to shed light on the woman’s condition living with the illness and treatment pathways they followed. There is lack of studies that focus on the entire illness experience for fistula patients and as such, this was the knowledge gap that our research sought to fill. This paper focuses on the period before obtaining fistula surgery, and documents women’s health seeking behaviour patterns during fistula illness in Kenya. We sought to answer the question: what patterns of health seeking do women with obstetric fistula display in their quest for healing?

The health seeking behaviour of patients in any particular illness is a reflection of their interaction with and their ultimate utilization of the health system [21]. An understanding of the health seeking behaviour of this group of patients is therefore important to improve health interventions to other patients with obstetric fistula. As such, qualitative methods of data collection and analysis were best suited to investigate both process and experience of fistula patients as they sought treatment for the illness.

## Methods

### Recruitment

The study participants were recruited from three fistula repair centres in Kenya—namely, Kenyatta National Hospital, Kisii Level 5 Hospital and Gynocare Fistula Centre—in 2013. Kenyatta National Hospital is a national referral hospital located in Nairobi city that holds at least one free fistula surgical camp annually to an estimated 400 patients. Kisii Level 5 Hospital is a public regional referral facility located in the southwestern part of the country and holds a free fistula surgical camp annually to about 50 patients. Gynocare Fistula Centre located in the North Rift holds an estimated 300 fistula surgeries throughout the year. We recruited patients who had undergone corrective surgery for fistula caused by labour and childbirth (obstetric fistula) as reported by the women from three hospitals. Women were approached by the researchers (AMK) and research assistants (ML and MO) during their inpatient stay and offered information about the study. Upon written informed consent, their permission to voluntary participate in the study was obtained. Only participants who voluntarily accepted participated in the study. A number refused participation though they were not obliged to give us their reasons. Those who refused to participate were not recorded.

### Ethical approval and consent to participate

Ethical clearance for the study was obtained from the University of Nairobi and Kenyatta National Hospital ethics and research committee (ERC) project number P618/11/2012. A standard written consent information sheet was used to explain the scope of the study and their role in participation, and the long term follow up nature of the study. Women gave verbal and written informed consent to participate in the study. They were made to understand that participation was voluntary and those who did not participate in the study would still receive care as planned by the clinical team. Only those participants who consented were recruited into the study.

### Consent for publication

This is not applicable since no photos, clinical images or videos of participants were collected nor are presented. Participants gave permission to use of a voice recorder in interviews and anonymous and un-identifying publication of the transcribed data resultant from the study. Participants’ anonymity has been guaranteed through labelling of their quotes using code numbers in place of names. In addition, their exact location is concealed and in its place, the name of the larger county. For instance, a participant labelled as (018_1, Siaya) means participant code number 018_1 lives in Siaya county.

### Grounded theory methodology

The study used grounded theory methodology [22]. Primary data was collected using interview guide. Narrative approach allowed participants to construct the events surrounding the index birth and context of occurring fistula illness, living with fistula and their health seeking behaviour. These interviews were audio recorded. The transcripts were imported and managed within NVIVO 10 software. This paper reports the findings of the first phase of the study that focused on the health seeking behaviour and treatment pathways of fistula patients. Due to space limitations, phase 2 of the study which focused on the topic of reintegration of fistula patients is reported in a separate article. We included women of various ages, level of education, geographical location, type of facility and number of children in keeping with deviant case analysis in grounded theory methodology to describe all possible conditions of fistula patients across three hospitals in Kenya.

### Reflexivity and positionality

AMK a female PhD student and registered nurse midwife conducted the interviews with the help of ML and MO, female research assistants. The research team had training in qualitative health research in conducting qualitative research and hospital ethnography. In hospitals, the data collection research team wore normal clothes, no uniform nor badge, presenting as nursing student researchers. This naïve positionality enabled us to reduce the power positions clinician researchers have over the participant and improved interactions with participants. The researcher assumption was that women did not want to go to hospital hence long decades with illness. Later, we discuss how this assumption was refuted, as women lay claim to an unresponsive system than their unwillingness to seek treatment.

### Data collection

(AMK) and trained research assistants (ML and MO) conducted the interviews in a hospital setting using a narrative guide in Kiswahili. Additional file 1 shows the narrative guide that was used in the study. The first broad question was highly exploratory allowing the participant to start from when they delivered the baby and the subsequent steps they followed. Subsequent probes and follow up questions then steered the conversation further. The interview guide is attached. Interviews generally lasted for forty to ninety minutes depending on the length of the participant and their style of narration. There were no repeat interviews on the topic of health seeking behaviour. Instead, participants were let to tell their narrative and probes used to ascertain any facts they may have left out. The audio recording was translated from Kiswahili to English then transcribed by AMK assisted by YB a trained research assistant. The researchers kept field notes, observing and recording any detail information to further refine the research and help in interpretation and analysis. Concerning participant checking, transcripts were not returned to participants for this phase of the study. The nature of their short hospital stay (maximum 9 days) meant any clarifications would be sought during the second phase of the study. Rather, thorough probes and follow up questions were used on the interview guide to ensure clear and complete information. In addition, during the initial interviews, some participants were asked if they agreed with the perceived meaning of their interview data.

### Theoretical sampling

Purposive sampling was used to recruit patients whose vaginal fistula had been caused by childbirth and had undergone vaginal fistula surgery. The systematic nature of grounded theory led to analysing data from the initial 6 interviews at the Gynocare centre where data saturation occurred at 30 interviews. However, an additional 5 participants were recruited to cater for loss to follow up that may occur in a 1 year follow up study (that was focused on reintegration outcomes). There was need for a flexible approach to sampling due to the rapid nature of fistula camps held just for a week annually at Kenyatta and Kisii. In these two centres we took similar cue from the first study site leading to 30 participants at Kisii and 48 participants at Kenyatta with an additional 8 to total 56. Data saturation in the last study site was obtained using a larger sample because the participant characteristics were less homogenous and their narratives had broad variations. The sample size was guided by existing research on number of participants in qualitative studies, variation across the three study sites, the need for ample numbers to cater for loss to follow up and sufficient numbers for theory development [23]. Finally, to further develop certain emerging categories into theoretical construct, theoretical sampling was used to pursue and interview participants, whose narrative contained the chosen phenomena to fully describe it, for instance, ‘staying home with fistula illness’ as a health action in the health seeking pathway of fistula patients.

### Data analysis

Grounded theory methodology following the classical Glaserian process of 3 stage coding – namely open, axial and selective axial coding - was applied [22]. One researcher (AMK) coded all data on health seeking behaviour, obtaining the emerging themes. Apriori themes were not set before interview and analysis. Open coding was conducted on initial 6 narratives where as many as 16 emerging themes (categories) and 67 subthemes were identified and their properties defined. Open coding generated first order and in vivo themes such as ‘I stayed with my illness’. Axial coding then linked the various categories with the subcategories. In selective coding, the findings were further integrated and refined into theoretical constructs. For instance, the invivo code ‘I stayed with my illness’ was further examined and fell under themes of living with fistula illness and the moral properties of suffering; as well as a strategy and (in)action in response to the illness. Selective coding of the transcripts led to the core category of health seeking behaviour pathway for fistula patients, that incorporates ‘staying with illness’ as a subcategory, alongside other core phenomenon such as ‘seeking the formal health facilities’. Eventually, more narratives dwelt on their experiences seeking formal health care making it a core phenomenon, whilst less and less instances of staying home arose. Another core phenomenon is the length of time spent living with fistula illness.

Throughout the study, we wrote memos that were case based and reflecting on what we have learnt in a particular interview. In addition, we wrote conceptual memos about an initial code or focused code, describing its meaning, comparing between data and asking questions that would need to be answered in further interviews. For example, a memo on the focused code ‘living with fistula’ stated:

“Themes of having psychological thoughts; social isolation; suffering overlap in the women’s narratives and allude to the moral properties of living with fistula. Although the women rarely refer to suffering directly, their narratives elicit feelings of *helplessness, sadness, emotional pain, shame* and at times *crying* during interviews. Occasionally, *suicidal thoughts* and feelings of (perceived) iminent danger of death from the illness permeate women’s narratives of their lives with obstetric fistula. These are properties that define their experience of suffering. At times, they refer to their illness as a 'problem' *shida* a term which in local Kiswahili dialect that refers to a situation in one’s life that needs help. Content describing women’s *suffering* closely follows or links with their isolated lives and the mental anguish they experience. Although counselling exists in varied form across the three sites, how much and at what frequency and style is it needed to undo the ill mental health women with fistula have undergone?”

Participants’ individual narratives were read and a pathway constructed. A composite pathway emerging from the narratives was then generated and the model drawn. The analysis yielded the seven key actors and likely actions in the treatment pathway of fistula patients. Living with fistula and seeking treatment took on a chronic illness career as presented in section 2.0. Many women wove their health seeking narrative with the difficulties, challenges, frustrations and disappointments of living with fistula.

While, to a researcher, an objective health seeking pathway should be devoid of other life events, to the women the context is inseparable from the experience. It is this rich and nuanced data that became the hallmark of our research that is presented to further illuminate these pathways. The narratives with fistula patients led us into a glimpse of their entire life story centred on the fistula illness.

Data saturation is defined as ‘the point in data collection and analysis when new information produces little or no change to the codebook’ [23]. On the other hand, theoretical saturation is assumed ‘once all main variations of the phenomenon had been identified and incorporated into the emerging theory’ [23]. Thus, data saturation may be obtained with fewer interviews than that required for theoretical saturation. For instance, Wilson, et al. used 66 interviews to develop a grounded theory on HIV/AIDS reconciling incompatibilities theory [24], whilst Mason and Harris used a sample of 114 in a study of environmental factors that influenced market orientation [25]. A heterogeneous sample of fistula patients, the broad research question and on a subject of health seeking behaviour where little was known beforehand necessitated a sample of 121 before we could reach theoretical saturation. Thus the composite pathway of fistula patients’ health seeking behaviour incorporated all the possible variations of this phenomenon.

**Fig. 1:**
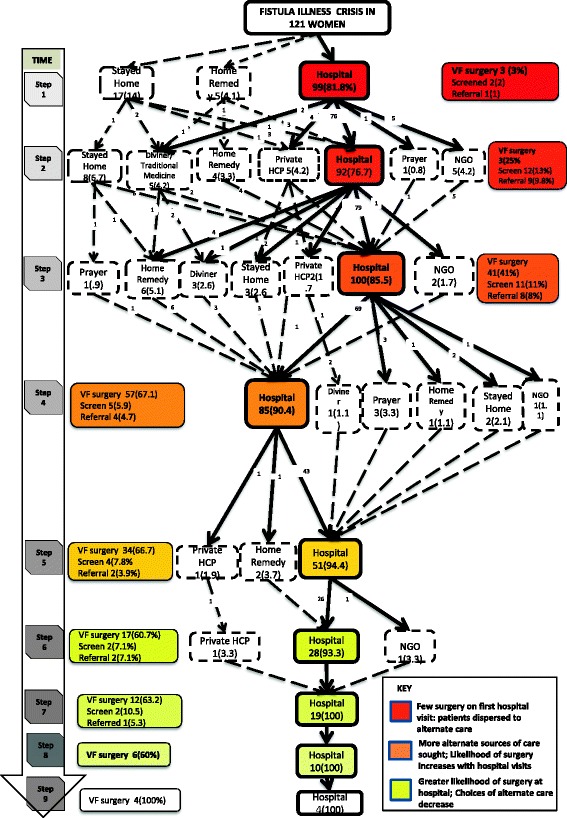
Composite Health Seeking Behaviour Pathway. A diagram illustrating the path followed by patients with obstetric fistula whilst seeking treatment

**Fig. 2:**
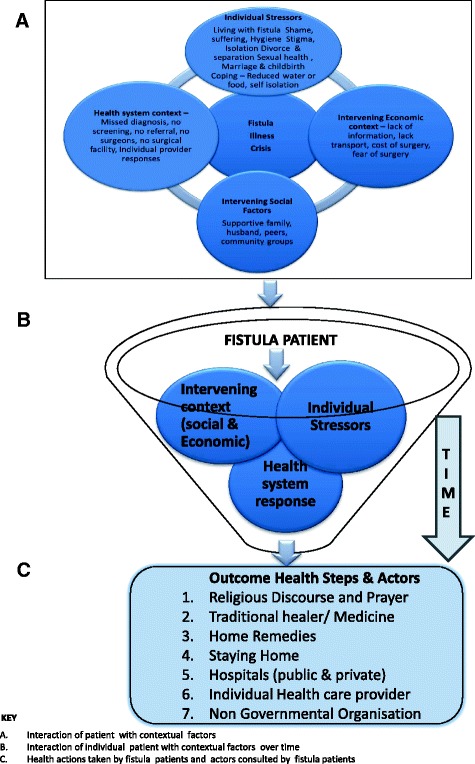
Conceptual Model of Health Seeking Behaviour. A diagram illustrating the key concepts in the health seeking behaviour of patients with obstetric fistula

The conceptual framework presented fits with the data and incidents described. Fit was obtained through constant comparative coding where one emerging concepts were checked and compared with other participants narratives in similar and varying contexts to verify whether they exist. Considering the findings presented in quotes and composite health seeking pathway in Fig. 1, the conceptual framework in Fig. 2 is adequate in reflecting the contextual issues surrounding fistula care in Kenya. The qualitative findings strongly suggest, for example, that the conceptual model fits the constructs of fistula as a chronic illness over time; seeking formal care in hospitals alongside alternative therapies; and the interplay of contextual social, economic, health system and individual factors that determine health actions of women with fistula illness.

## Results

### Background characteristics

A total of 121 participants (women with obstetric fistula) gave their narratives of the experience of health seeking behaviour during fistula illness. These narratives were used to construct the composite pathway. The demographic characteristics of 2 women whose narratives are included in the composite pathway were not obtained, and consequently 119 women’s are presented. The participants’ mean age was 33.2 (17 to 62 years). The mean age at fistula development was 23.2 years. Women generally had low level of education, with 12.6% having no formal education and a further 54.7% had obtained primary education only. About a third of the women had no surviving child and half had developed fistula during their first pregnancy delivery. The participants described long episodes of labour at home under unskilled birth attendance mean 48 h (Table 1).

**Table 1:** Participant characteristics

Variable		Frequency (n)	Percent (%)
Hospital Treated (*N* = 121)	Gynocare	35	29.4
	Kenyatta National Hospital	56	45.4
	Kisii Level 5 Hospital	30	25.2
Age in Years at time of study (N = 121) Mean 33.2 years Median 31.0 Mode 28 Range 40 (17–67)	15–19	9	7.6
	20–24	15	12.6
	25–29	27	22.7
	30–34	20	16.8
	35–39	13	10.9
	40–44	9	7.6
	45–49	7	5.9
	50–54	8	6.7
	55–59	3	2.5
	60–64	4	3.4
	65–69	1	0.8
	Don’t know	3	–
	Not determined	2	–
Age in years at onset of fistula (N = 118) Mean 23.2 Median 22 Mode 18 Range 44	10–14	10	8.4
	15–19	30	25.2
	20–24	31	26.1
	25–29	26	21.8
	30–34	5	4.2
	35–39	10	8.4
	40–44	2	1.7
	45–49	1	.8
	55–59	1	.8
	Don’t know	1	2.5
	Not determined	2	
Level of Education (*N* = 119)	None	15	12.6
	Primary 1–4	9	7.6
	Primary 5–8	56	47.1
	Secondary	33	27.7
	College	6	5.0
	Total	119	100.0
No. of Surviving children (N = 118) Mean 2.0 Median 1.49 Mode 0 Range 9	0	35	29.7
	1	26	22.0
	2	19	16.1
	3	11	9.3
	4	13	11.0
	5	5	4.2
	6	4	3.4
	7	3	2.5
	8	1	0.8
	9	1	0.8
	Total	119	100
Order of pregnancy when fistula occurred (N = 118)	1st	61	51.7
	2nd	24	20.3
	3rd	9	7.6
	4th	11	9.3
	5th	5	4.2
	6th	2	1.7
	7th	3	2.5
	8th	1	0.8
	9th	1	0.8
	10th	1	0.8
	Total	118	100
Total Hospital Visits before surgery (N = 119) Mean 4.13 Median 4 Mode 4 Range 9	1	3	2.5
	2	20	16.8
	3	23	20.2
	4	29	24.4
	5	21	18.5
	6	6	5.0
	7	8	6.7
	8	4	3.4
	9	3	2.5
	Total	119	100.0
Total No. of Surgeries (*N* = 117) Mean 1.71 Median 1 Mode 1 Range 5	1	78	66.7
	2	15	12.8
	3	12	10.3
	4	6	5.1
	5	4	3.4
	6	2	1.7
	Total	117	100
Ever had fistula surgery prior to this one? (N = 117)	Yes	41	35.0
	No	76	65.0
	Total	117	100.0
Where did you deliver the baby? (N = 119)	Home	23	19.3
	TBA (Traditional Birth attendant)	4	3.4
	Hospital	92	77.3
Time in years lived with fistula Mean 8.9 Median 6.0 Mode 7.0 Range 39.0 (0.8–39.08)			

### Emergent themes in health seeking behaviour

#### Composite pathway of health seeking behaviour

In order to understand the health behaviour patterns of women with obstetric fistula, it was vital to collate a pattern of care consultation pathways. The pathway (Fig. 1) demonstrates participants’ likely movements since symptom recognition to the point we meet them at a fistula repair centre [26].

These results are particularly helpful in describing in totality the landscape of fistula care in Kenya. The onset of fistula illness’ symptoms is a crisis that triggers help seeking amongst women to regain normalcy. Although there are many symptoms of fistula, the women recognize most easily the urine and stool incontinence. The symptoms are at times not only recognized by the participant but close family members. Throughout the article, we provide data in form of quotes obtained from women across different age ranges and from women with or without children. For instance, a typical narrative starts with symptoms around childbirth:

When they pulled the child [Assisted Vaginal Delivery] that day water [urine] started to come out together with faeces. Now, the faeces were coming out as if it is watery. And the urine! I sit like this when I get up… Even if you have eaten *ndengu (lentils)* it comes out that way. When I drink tea it pours like that. And they come out together. (018_1, Siaya).

The onset of visible symptoms of urinary and faecal incontinence is a mark of abnormality to the patient. The onset of fistula illness and patient’s recognition of symptoms are a crisis that the individual resolves through staying home with illness; reaching out to family and friends for support and / or reaching out to the formal and informal systems for treatment. ‘I stayed with my illness’ was a common phrase that was expressed by participants in describing the sequel of events that followed recognition of symptoms of fistula illness. First, they used it to denote the range of experiences of living with fistula illness and the challenges this posed. These experiences included having to deal with.

Now it had reached a point I felt like strangling myself because if I cannot go to my own home, I can go near my own people, I can’t go near anyone because of that problem, I now used to feel when I reach a point and take away my life. I used to say it is better to die because I couldn’t even go to the market. (004_2, Kiambu).

Secondly, staying home was a step in the health seeking trajectory, as demonstrated in the quote below:

I just persevered with my problem. Urine would flow a lot. At this time I would find it difficult sit on a chair. Even when my friends came I would not agree to sit. I would be asleep but inside was water. Then the urine started flowing less and I started wearing *always* [sanitary pads]. I could now walk around without anyone noticing my problem. I persevered with it without telling anyone. I did not seek for help at all because I did not want anyone to know my problem. (004_3, Kisii).

As demonstrated in the composite pathway, most women did not receive corrective surgery at the first point of contact with the formal healthcare system. A paltry 3 women did receive Vesico Vaginal Fistula (VVF) surgery at their first visit to hospital (step 1), and another 3 screened and referred. The remaining 93 women are not attended to appropriately in their first step at the hospital. A similar impediment faced the 76 who come back to a hospital as the second step, only they are joined by more who had opted to stay home or seek home remedies. Others however stayed home or tried prayer, alternate home remedies and traditional medicine and returned to the formal health system much later in their treatment seeking trajectory. An exemplar excerpt explaining multiple hospital visits, referral and trying alternative medication is derived from this participant’s narrative:

I had gone to lancet building, and I was told that I will go for surgery in Nairobi west and it was too difficult because it was too expensive, I was to pay one something, one hundred and something thousands [about 1000US Dollars]. From there I went to Gatundu general. First they did not know what the problem was, then later I saw a gynecologist who knew what the problem was. But so they used to postpone the surgery… then there was this other time that I went to the hospital, a place where they sell medicine here in Eastleigh they are Indians. I was given other stones which you insert and a certain powder that was to be mixed with water then and it becomes paste then you insert inside [vagina]. That was the thing that brought this infection. (026_2, Nairobi).

#### The role of formal hospitals

We found that, first, contact with hospital was no guarantee of treatment for obstetric fistula thus many participants had multiple hospital visits to different facilities over decades without treatment. Secondly, unsuccessful surgery led to the women staying home for long periods before they attempted another visit to hospital. Third, women consulted with alternative health care providers like diviners, traditional healers, nongovernmental organizations or private healthcare providers.

A participant who had lived with the illness for 34 years had obtained fistula surgery within two years of illness onset but it was not successful. She went to different hospitals then stopped more efforts to obtain surgery when she realized it was expensive. She then sought to use herbal medicine, staying home, home remedy and prayer. She explained:

There [hospital] they examined me and said my bladder was destroyed. They told me to go and eat well for three months and then I go there so that they can try treating me. So when I saw that the money required at Kindu Hospital was a lot, I went to Russia hospital. I was treated there but I did not heal at all. The urine continued coming out. They then told me to go back there but I was afraid. I went to another hospital in Kiambu called Nazareth hospital. I was treated there and was not healed. So since then I have stayed in the house; I don’t go anywhere but tolerate the problem since that ‘79 until now, 2013. That’s when I heard the radio advertising that there will be a doctor here who will come and help the women who continue to have urine coming out. That’s when I tried and came here and I have been treated (030_3, Kisii).

The interplay of unsuccessful surgery, lack of money and alternative medicine keep women staying with fistula in the communities. Indeed women expressed frustration with multiple surgery and unsuccessful repairs. But the multiple visits for treatment were a series of costly visits even though they could not afford it. In this instance they gave up and learnt to tolerate their situation.

A main impediment to women access to hospital was poverty and lack of money to pay for procedures related to or for the fistula surgery. For instance, a participant who lived with fistula illness for thirty years described lack of money as an impediment to treatment.

I could not get money … because we were told these things require money. I was operated on and they told me when I go back I go with money. And I was told it was a lot of money and I said where will I get all these money, even if it is ksh100, 000 where will I get it from? I am from the rural areas and there is no one who can give me. So I said let me just stay if it was written for me like that I will stay and die like that. So when this child heard about it he told me, ‘mother, come so that you can be treated’. (033_2F, Siaya).

The lack of proper information regarding fistula among health care providers is a shocking reality represented in the face of participants who go to hospital numerous times without getting proper examination, diagnosis and reparative surgery or referral. It is one of the circumstances that women meet in hospital before they take other steps to the other alternatives in the health seeking pathway other than hospital. A typical narrative that demonstrates this is the story of a participant who kept coming back to the same facility five times before she was informed that her condition could not be treated there. This then meant she had multiple contacts with one facility without definitive treatment, and ultimately she was not offered an alternative referral as to where she would get proper treatment. She went home and ‘stayed with her illness’ as she narrated:

In Kakamega I received the operation [caesarean section]. So they told me that it was dirt coming from the abdomen stomach and it will just be over. When I went back home the problem continued. They gave me another appointment. So when I went for that appointment, there was no doctor. And they gave me another appointment. I went and they told me, ‘you could have been operated on in the theatre but there is no water. Come back’. And they gave me another appointment. I went back on that date, and they told me there was no cotton wool to do that job. I went back home. They gave me another appointment. So I went and they put me in the ward. I stayed in the ward for two weeks. Then they said, ‘when we examine you, we see that you are destroyed badly, and we cannot’, so there they said the truth. […]. ‘So now I will go back home?’ Yes. I went back home. I went to Mission hospital and they told me to go back to Kakamega, ‘we don’t treat people who are like this’. I said I have gone many times to Kakamega and they shocked me this last time when they told me that they cannot treat the illness. So I went back home and stayed. I thought if they refused to treat me in Kakamega, I am destroyed badly and it’s not possible. Now I said, ‘I have tried Mission hospital and they have taken me back there, it is like it has failed’. I went home and stayed. (036_1F, Kakamega).

She is not alone in the narrative of health care providers missing fistula diagnosis, or failing to refer patients appropriately with enough information about their condition and where they can obtain treatment.

Another significant finding of this pathway is that women undergo multiple surgeries in their quest for healing. Some had the surgeries at different hospitals and VVF camps, some by same or different surgical teams. Thus, from the examples shown above, the multiple visits are characterized by a health system that did not have enough equipment and expertise to treat the illness, at times failing to refer the women to facilities that would. Secondly, if money was required for tests, the participant was not likely to afford the test. Third, when a participant is afraid to go back to hospital she is not likely to get treated. The frustrations represented in the pathway to accessing treatment may have led to some women ‘just staying at home’.

#### Conceptual framework of health seeking behaviour

The numerous factors presented in the composite pathway can be summed in a conceptual framework that depicts the contextual issues surrounding fistula treatment in Kenya [26]. They include individual stressors, economic factors, health system factors and social factors. The interplay between these factors results in the health actions and consultation of actors in care of fistula patients, namely religious discourse and prayer, traditional healers and medicine, private healthcare providers, private clinics, Nongovernmental organizations, or staying home and use of home remedies. The conceptual model is presented in Fig. 2 below [26].

A participant who used traditional medicines narrated:

Others were telling me that with this illness I shall just take traditional medicine it shall end. I took the medicines there wasn’t anything, I was taken to the witch doctor there wasn’t anything I said eey maybe this is my death. I stayed until 2009. (009_2, Homa Bay).

Over time since onset of fistula illness crisis to surgery, the obstetric fistula patient experiences broad contextual and intervening factors that determine each health action they will take. The intervening factors overlap to produce varied effects seen as steps in the health seeking behaviour patients with obstetric fistula.

The intervening context includes economic and environmental factors such as availability or lack of information, transport, cost of surgery, use of mobile phones to link the fistula centres contact person; social factors such as presence of supportive family especially the husband, peers, and *chama* (community groups); Health system factors such as missed diagnosis, no screening, no referral, surgeons, surgical facilities, individual provider response to fistula patients influence their health steps/ actions.

She told me there was a hospital called Gynocare and if you go there and talk them they might assist you. So she gave the phone number of one of the ladies here … and when I called him and explained to him about my problem he told me that at the moment that all the beds were occupied but he was going to see on how they go and see on how they were going to assist me. When I arrived he sent the madam called **** when I had reached at the reception and then that lady brought me here where I met the doctor on that day and he told me I had to be sewed operated on. (022_1, Kakamega).

Ultimately, intervening contextual factors – economic, social and health system – interact with the individual stressors to determine their sequential progress and steps in the health treatment seeking pathway. Although individual patients may live in the same contextual environment, their individual stressors uniquely impress on how they respond to and reach out for help in this crisis.

First, individual stressors include the entire experience of living with fistula illness. Implicit in this is the shame, stigma and isolation and the entire moral experience of the disease. Whether a patient discloses and seeks help for fistula illness depends on the strength and quality of their moral experience of the illness. Further, disclosure relies on the social support they obtain to seek treatment in their quest for healing. One participant explained thus:

Secondly, hygiene challenges posed to patients hinder them from accessing public transport to seek treatment. They are caged in their own lives avoiding public scrutiny and shame. In instances where transport is provided by a Non-Governmental Organisation (NGO) to hospital, women are more confident than if they use public transport. For instance,

Thirdly, individual stressor is divorce and separation that plays out both on the social and individual stressor role. Divorced or separated women have little support from spouse in their health seeking trajectory, especially when money for transport and hospital costs is needed. On the contrary, women who are supported by their spouse interact better with health systems. Hygiene challenges and lack of any surviving children contribute to separation and divorce, leaving the woman without the support of the husband as in this participant’s narrative:

It was very bad because when we stayed, my husband wanted a child and I didn’t have any so he married another wife and am alone. He wronged me but God is there… You see I had this illness so he couldn’t come to where I was [smell] and he wanted a child so he went and looked for another wife and got married. (054_2, Nyandarua).

On the contrary, participants whose husband was supportive psychologically and financially coped with the illness better, as in the case of 004_2 who said ‘My husband tried as he could look for a job so that I get the money for going to the hospital… He was telling me just not worry one day I will get treatment’.

Finally, individual coping mechanisms like self-isolation are stressors. If occurring in a woman who does not have sufficient information on the illness, then she will not be able to get social support nor contact the health system and obtain surgery in time. This was particularly true of participant 004_3 who said ‘I persevered with it without telling anyone. I did not seek for help at all because I did not want anyone to know my problem’. (004_3, Kisii).

Two core issues that drive health seeking by women are hygiene concerns and desire to regain normal reproductive function. There are however enablers and disablers to treatment of fistula patients at the individual context and health system level as described above.

### What do the findings mean for women’s health seeking behaviour?

We present the health seeking behaviour of fistula patients using a conceptual framework grounded on the narrative data that sufficiently takes into account the context surrounding fistula care in Kenya. A typical storyline of health seeking behaviour during fistula illness depicted in our study is that of sequential pathways with initial many visits to hospital getting few surgeries. Fistula therefore becomes a chronic illness that women have to live with. Living with fistula entails moral properties of suffering with generally long pathway to care that did not always guarantee access to care. There is a deviation from normal and women strive to regain normalcy in their physical health, social acceptability, marriage and economic independence.

Women’s realization of symptoms of both rectovaginal and vesicovaginal fistula sets off an account of a pathway that did not always guarantee access to care. The women meet a health system unresponsive to needs of women with few fistula experts, missed diagnosis, lack of pertinent information and logistical support. Further, the women themselves may not be motivated to undergo surgery, for fear of death and due to poverty. Lack of social support, divorce and separation add to the other stressors in the process of health seeking. Few women experience the ideal enablers of healing and regaining normalcy, namely, correct information through radio, access and linkages to care through mobile phones, and most vital surgical facility and expertise at a cost they can afford.

A typical narrative of health seeking for a fistula patient entails sequential pathways where she moves from place to place, person to person seeking treatment. Initially, many visits to hospital get few surgeries, owing to lack of facility, lack of medical team with surgical expertise at the hospital or high cost of the surgery. What follows however, for this and many other similar stories is daunting. Fistula therefore becomes a chronic illness that women have to live with, attempting multiple times to regain normalcy.

The composite treatment pathway is characterized by a lengthy time living with illness, multiple visits to different actors and a core narrative of seeking the formal health facilities (denoted as hospital) in the quest for healing. Emerging themes examined in this pathway namely, cost of surgery, unavailability of surgery; missed opportunities to diagnose and refer patients are amongst the key stumbling blocks that women with fistula face in seeking treatment at the hospital. Consequently, other health steps or actions include trial of home remedy and traditional medicine in trying to heal their condition. Granted, patients’ encountering a system that does not cater for their needs is frustrating and leads to long decades of living with fistula illness. Further, not knowing what illness it is and how it is treated (unawareness) further delays women from obtaining surgical treatment. Treatment pathways are impeded by lack of knowledge, transport, money; multiple hospital visits, referrals, and cost of surgery.

Patients’ perception of their experience to screening and treatment may add to a perception that the system is weak and inefficient in treating fistula survivors; and by word of mouth encourage women to refer or not to refer other women who have the illness for treatment. Due to dysfunctional health system, obstetric fistula becomes a chronic stigmatized illness with the hospital as a key disperser of patients to other health care choices as demonstrated.

Ideal cases of obtaining fistula surgery at the first step of seeking treatment in hospital are rare, with only 3 out of 99 women who sought care at hospital at the first step undergoing corrective surgery for either Recto Vaginal Fistula (RVF) or Vesico Vaginal Fistula (VVF). The proportion accessing surgery increases upon multiple visits at the second and subsequent steps at hospital visits. A close scrutiny of the pathways reveals that initially, women stay home, as time advanced, more women procured surgery. The extremely slow cases are represented by women who had five or more visits to the hospital and got treatment having lived with fistula for many years.

## Discussion

### Health seeking behaviour and health system (un)response

There is scarce research concerning the health seeking behaviour patterns of women with obstetric fistula. By the time of this study, no study existed that wholly examined the health behaviour patterns of fistula patients beyond reporting the time spent before surgery [18]. However, existing research has illuminated some of the components of health seeking behaviour similar to our study.

A recent study by Maulet, Berthé [27] reported that obstetric fistula illness may be viewed as a chronic illness owing to the length of care process, limitations of surgery and the persistent physical and moral suffering [27]. Another qualitative study spanning 18 months in Mali and Niger, established the need for long term care, and tertiary prevention measures, but acknowledge that the holistic approach to care, prevention-treatment-reintegration axis may be feasible within standalone fistula repair centres and international NGOs but a challenge to the national referral hospitals that are biomedical oriented and overwhelmed with emergencies and other referrals [28]. In our study, women report multiple visits to hospital for surgery and less than half had repeated surgeries. However, our study participants were not resident in hospitals as they awaited the next surgery, as opposed to the Mali study [27]. Granted, Maulet, Berthé [27] contribution highlights the long care process of fistula patients, suggesting that surgery is not a one-time episode as women undergo multiple surgeries. This care process after proper diagnosis may be long [27]. In our study, about half the women had undergone one previous surgery by the time of the study, confirming the long term nature of fistula care.

Although obstetric fistula is not considered to be amongst chronic illnesses [29] the length of time spent with illness and illness trajectory mimics other chronic illness. This emerging concept has been alluded to by other authors [31]. However, based on the findings of this study we contend that the chronicity of fistula illness is beyond the time spent during the care process as suggested by Maulet., et al. [28]. The total time lived with fistula illness is the first clearest indicator, in the biography of women who suffer this condition, that obstetric fistula is a chronic illness. Secondly, depending on the surgery outcome, irreparable fistula presents the woman with the possibility of living with fistula illness for the rest of her life. Those who encounter other forms of corrective surgery like urinary diversion permanently live with the aftermath of fistula illness through the changed function of their bowel movement from the ‘normal’. Infertility is another fistula illness symptom that lingers after surgery, along with traumatic psychological moral experiences. Obstetric fistula illness is thus in this sense, a chronic illness with dire consequence on the woman and her family. Further, women who have undergone fistula surgery are required subsequent pregnancies through caesarean section.

Regarding time lived with fistula illness, a recent study in a western Kenya hospital indicated that 35% of the women had experienced incontinence for one to five years and a further 19% for over five years [18]. Similarly Yeakey, et al. [30] report a period of up to 20 years living with the condition in a qualitative study of the lived experiences of women with obstetric fistula, suggesting a long term nature of suffering fistula illness. Our study unravelled similar and more dire findings; of women who had lived with fistula illness for up to 43 years. The chronicity of fistula illness is suggested, first in the longevity of the illness and secondly in the way the woman’s life is disrupted and the normal as she knew is halted.

Wall [31] has suggested that obstetric fistula is a neglected tropical condition. Our findings support this author’s position, and even go further to demonstrate that neglect of obstetric fistula patients has led to the chronicity of fistula illness. Through the pathways, we demonstrate a health system which often disperses women seeking treatment to other actors in the field who are ill equipped to treat fistula. Although hospital is a popular choice as steps of action in women’s pathways, it does not always obtain for them the treatment and healing they seek. Further, our findings suggest a systematic failure in the health care system to cater for the needs of women who present with obstetric fistula at the hospital. As such, the hospital is a key disperser of women to other forms of care providers; yet it is the most popular step taken by women when they realize symptoms of bowel and urinary incontinence.

Our study introduces new explanations to what characterizes the chronic nature of fistula illness. As such, the lack of a broad range of other studies on the health seeking behaviour of fistula patients limits chances of comparison. For instance, we report 7 key actors on the pathways chain including the hospital and health clinics, private health care providers, traditional medicine and diviners, religious discourses and prayer, Non-Governmental Organization and home remedies and staying home the participants can take. We report for the first time a broad range of multiplicity of actors in fistula care. Some studies in Tanzania a [32] and Uganda [33] have reported the use of religion as coping mechanism while in Ghana [34] reliance on traditional medicine has been reported. A prolonged lag time between onset of fistula illness and first hospital visit, was related to lack of awareness and few fistula repair centres and unacceptable modern care [11].

The numerous hospital visits without proper referral and surgery is for the first time focused on in our study. However, seeking of other avenues of healing including traditional medicine, prayer and healers are phenomenon reported elsewhere in studies of health behaviour and therapy management for malaria in Kenya [35]. Similarly, Mwini-Nyaledzigbor, Agana [34] reports use of traditional herbs to treat fistula in a study conducted in Ghana.

In our study, the innovative use of mobile phone communication was a good enabler to women’s reaching fistula repair facilities. These findings complement those of Fiander, Ndahani [36] in the innovative usage of mobile phones to link women with fistula to care in providing information and transport costs. The Comprehensive Community Based Rehabilitation program in Tanzania used mobile phones to transfer money to ambassadors who then assist the patients to cover transport costs while they seek treatment. This improved the number of referrals for fistula surgery combined with free treatment was owed to efficient mobile communication, training of the ambassadors and eliminated cost of transport [36]. In our study however, the mobile phone was used to contact the regional representative concerning scheduled dates in one of the dedicated fistula centres; and later during follow up care after surgery. This innovative use of commonplace means of communication to provide accurate information concerning an individual woman’s care is sustainable and offers possible exploration in future.

Enablers of health seeking behaviour should be emphasized on basing on women’s treatment experience. For instance, information about treatment services and access to treatment to patients living with obstetric fistula through radio advertisements, mobile phone contact by regional representative, and contact by health care providers and referral by health care provider are possible avenues of improving the experience and shortening the women’s treatment seeking pathway.

### Recommendations

Based on the study findings, we make the following recommendations. Firstly, there is a need to treat women with obstetric fistula promptly; given the immense suffering the illness poses and the pathway through which they seek care can be improved. This treatment should be by qualified fistula surgeons. Secondly, in view of a lot of women living with fistula for decades, the current treatment is a little too late and early referral and treatment ought to be available, to decrease backlog and treat all new cases. In this regard, building the capacity of the health system and personnel through training of midwives and other health personnel on the proper information regarding fistula prevention, recognition of symptoms and management and improving surgical facilities are priority areas to be focused on by policy and resource allocation. The members of general communities should be informed about obstetric fistula prevention, signs and symptoms and where to seek treatment when the illness occurs and how to support patients with obstetric fistula and post- surgery period.

### Study limitations

The participants in this study had undergone fistula surgery and no information was obtained from women who didn’t access the formal hospital for surgery.

## Conclusion

The recognition of symptoms of fistula – urinary and faecal incontinence - triggers women in a crisis that they have to resolve through ensuing health actions. Women generally follow linear pathways in seeking treatment for fistula with multiple actors in their trajectory being hospital, traditional healing, and home remedy or actions such as just staying home.

Contact with the hospital does not guarantee treatment for obstetric fistula. Long decades are characterized by multiple hospital visits devoid of proper treatment. Lack of knowledge of the condition, lack of transport, shame and hygiene challenges of using public transport, lack of money to pay for treatment and failure to locate hospitals conducting fistula surgeries hinder the health seeking behaviour of fistula patients. Referral and undergoing two or more corrective surgeries are common in the treatment pathway. These increase the number of visits and length of time lived with illness. Stigma may also delay women from disclosing and seeking treatment. The pathways model is most suitable to investigate health behaviour patterns of fistula patients with qualitative methods giving a deeper understanding of women’s health seeking behaviour.

## Additional file

Additional file 1: Narrative guide. The set of questions used to guide the narrative interviews with the study participants. (DOCX 64 kb)

## Abbreviations

ERC: Ethics and Research Committee
HIV/AIDS: Human Immunodeficiency Virus/ Acquired Immune Deficiency Syndrome
NGO: Non-Governmental Organisation
RVF: Recto Vaginal Fistula
VVF: Vesico Vaginal Fistula
