# Supplementary material for: ‘I stayed with my illness’: a grounded theory study of health seeking behaviour and treatment pathways of patients with obstetric fistula in Kenya
Source: BMC Womens Health. 2017 Sep 29;17:92. doi: 10.1186/s12905-017-0451-6 (PMC5622500; doi:10.1186/s12905-017-0451-6)
Supplement: Additional file 1: — Narrative guide. The set of questions used to guide the narrative interviews with the study participants. (DOCX 64 kb) [file 12905_2017_451_MOESM1_ESM.docx]

**HEALTH SEEKING BEHAVIOUR AND REINTEGRATION OF PATIENTS WITH OBSTETRIC FISTULA IN KENYA**

**Informed Consent Form Patients**

**Principal Investigator**: Anne M. Khisa

**Supervisors:** 1). Dr Grace Omoni

2). Professor Isaac K. Nyamongo

**Sponsors Name**: Consortium for Advanced Training and Research in Africa (CARTA)

**Part I:** **Consent Information Sheet**

**Introduction**

Good morning/ afternoon. My name is Anne Khisa. I am a student at the University of Nairobi and as part of my studies I am carrying out a study on obstetric fistula. I would appreciate if you agree to participate in this study. Before you decide on whether to join the study or not, you will be provided with information regarding the study, a chance to ask questions and availed with a copy of this information sheet to keep.

**Why is this study being done?**

The study seeks to investigate the kind of help that women seek while suffering from obstetric fistula and how they settle back into their communities after surgery. You are being asked to join the study as a patient who has undergone surgery for obstetric fistula.

**What is expected of you?** The study is intended to continue up to one year from now and will require two interviews. If you accept to be recruited in the study, you are expected to be available for a follow up interview. The researcher will therefore visit you at your home in a year from now. The study is being conducted in three hospitals in Kenya.

**Are there any risks?** If you join the study you may feel embarrassed, worried or anxious when answering questions from the study. There is no physical risk anticipated by participating in this study.

**What benefit can you expect?** While the facts we obtain from study might not help you directly, by participating, you provide useful information that will help treatment programs to provide care to patients with obstetric fistula in a better way.

**Will you be paid to be in the study?** The study does not offer any monetary or material benefits to participants.

**You may refuse to be in the study.** You are free to decline joining the study or to withdraw from this study at any point, and in such a case no penalty or disfavour will be shown towards you.

**Confidentiality** The information you give will be treated confidentially and your true identity shall not be revealed. The study does not offer any monetary or material benefit to the participants.

**Withdrawal from the study** You are free to withdraw from the study at any stage and are not bound to provide any reasons. During interviews, it is your right to stop the interview at any point in time or decline to answer some of the questions

**Do you have any questions?** Please feel free to ask any question you may have concerning the study.

**Contact Information**

Should you need to clarify your rights as a study participant please contact Professor Guantai, Chairman, KNH/UON Research Ethics committee, Tel 020 726300 P.O. Box 20723-00202 Nairobi, Email: knhuonerc@gmail.com

Or contact Dr Grace Omoni, Director School of Nursing, University of Nairobi, on Tel. 0202711250 P.O Box 19676 – 00202 Email: omonigrace@hotmail.com

Should you need any help or information regarding the study contact the researcher, Anne Khisa, Tel. 0724 348 661, P.O Box 14670-00100 Nairobi, Email: annekhisa@gmail.com

**Part II: Certificate of Consent**

***Research participant***

I have been invited to participate in the study on obstetric fistula in Kenya. The foregoing information has been read to me. I have had the opportunity to ask questions about it and have been answered to my satisfaction. I hereby declare that I have voluntarily opted to participate in the study.

Signature: __________________________ ***OR*** Thumbprint

Date: ______________________________

Time: ______________________________

***Researcher***

I have accurately read out the information sheet to the potential participant, and to the best of my ability made sure that the participant understands that the following will be done:

1. The information given shall be handled in a confidential manner
2. Their true identity will not be revealed
3. Their freedom to withdraw from the study will be guaranteed and no disfavour will be shown to them in case they decide to withdraw.

I confirm that the participant has had opportunity to ask questions about the study and have answered her correctly. The participant has freely and voluntarily accepted to participate in the study and has not been coerced into giving consent. The participant has been provided with a copy of this informed consent form.

Interviewers Name:_______________ Signature:__________________ Date: ______________

Time: _________________________ Participant Code No. _______________________

**TABIA YA KUTAFUTA AFYA NA KUWAUNGANISHA NA JAMII WAGONJWA WANAOGUA FISTULA ITOKANAYO NA UZAZI NCHINI KENYA**

**Fomu Ya Ridhaa Wagonjwa Wanaougua Fistula**

**Mkuu wa uchunguzi**: Anne M. Khisa

**Wasimamizi:** 1 Dr Grace Omoni

2 Professor Isaac K. Nyamongo

**Wadhamini:** Consortium for Advanced Training and Research in Africa (CARTA) *muungano wa mafunzo ya juu na utafiti katika Afrika*

**Sehemu ya I: Karatasi ya Maelezo ya Idhini**

**Utangulizi**

Habari za asubuhi / mchana. Jina langu ni Anne Khisa. Mimi ni mwanafunzi katika Chuo Kikuu cha Nairobi na kama sehemu ya masomo yangu mimi ninafanya utafiti juu ya fistula ya uzazi. Kabla ya kuamua iwapo kujiunga na utafiti au la, wewe utatapewa taarifa kuhusu utafiti huo, nafasi ya kuuliza maswali na utapewa nakala ya karatasi hii habari ya kuhifadhi.

**Kwa nini utafiti huu unafanywa?** Utafiti huu unalenga kuchunguza aina ya msaada wanawake hutafuta wakati wakiwa wagonjwa wa fistula itokanayo na uzazi, na jinsi wanavyorudi kukaa katika jamii zao baada ya upasuaji. Unaombwa kujiunga na utafiti huu kama mgonjwa ambaye amefanyiwa upasuaji kwa ajili ya fistula ya uzazi.

**Yale yanayotarajiwa kutoka kwako:** Utafiti huu unaazimiwa kuendelea hadi mwaka mmoja kutoka sasa na utahitaji kushiriki mahojiano kwa mara ya pili siku za usoni. Ikiwa wewe utakubali kujiunga na utafiti huu, unatarajiriwa kujitolea muda wako wakati wa mahojiano ya ufuatiliaji kwa kipindi cha mwaka miezi tisa hadi mwaka mmoja ujao. Hivyo basi, mtafiti atakutembelea nyumbani kwako mwaka mmoja kuanzia sasa. Utafiti huu unaendeshwa katika hospitali tatu nchini Kenya.

**Je kuna hatari?** Unapojiunga na utafiti huu, unaweza kuhisi aibu, wasiwasi wakati ukijibu maswali ya utafiti huu. Haitarajiwi kuwa kuna hatari zozote za kimwili zinatokana na kushiriki katika utafiti huu.

**Ni faida gani unaweza kutarajia?** Habari tutakazopata kwa utafiti huu haziwezi kukufaidi moja kwa moj. Hata hivyo, taarifa hizi muhimu zitasaidia programu za matibabu kutoa huduma kwa wagonjwa wa fistula ya uzazi kwa njia bora zaidi.

**Je utalipwa kwa kushiriki katika utafiti?** Utafiti huu hautoi faida yoyote ya fedha au vifaa vyovyote kwa washiriki.

**Unaweza kukataa kushiriki utafiti huu.** Una uhuru kukataa kujiunga na utafiti huu au kuondoka kutoka utafiti huu wakati wowote, na katika hali kama hiyo hakuna adhabu au karaha itaonyeshwa dhidi yako. Kumbuka ya kwamba una uhuru kuyaacha mahojiano haya wakati wowote na haitakulazimu kutoa sababu zako za kuondoka. unawezapia kukataa kuyajibu baadhi ya maswali wakati wa mahojiano.

**Usiri.** Habari utakayotoa itashughulikiwa kwa siri na utambulisho wako wa kweli hautafichuliwa.

**Je una maswali yoyote?** Tafadhali jisikie huru kuuliza swali lolote unaweza kuwa nalo juu ya utafiti kabla ya kunipa idhini yako.

**Maelezo ya mawasiliano**

Je, unahitaji maelezo kuhusu haki yako kama mshiriki katika utafiti huu?

Tafadhali wasiliana na Profesa Guantai, Mwenyekiti wa Kamati ya Maadili ya Utafiti KNH/ UON Nambari ya simu: 020 726300 ext. 44102, Sanduku la posta: 20723-00202 Nairobi, Barua pepe: [knhuonerc@gmail.com](mailto:knhuonerc@gmail.com)

Unaweza kuwasiliana na Dr Grace Omoni, mkurugenzi wa shule ya uuguzi, Chuo kikuu cha Nairobi, nambari ya simu 0202711250, Barua pepe: omonigrace@hotmail.com, sanduku la posta 19676 – 00202 Nairobi,

Je, unahitaji msaada au habari kuhusu utafiti huu? Tafadhali wasiliana nami mtafiti Anne Khisa Nambari ya simu: 0724 348 661, Sanduku la Posta: 14670-00100 Nairobi,
Barua pepe: [annekhisa@gmail.com](mailto:annekhisa@gmail.com)

**Sehemu ya II: Hati ya Idhini**

Nimealikwa kushiriki katika utafiti juu ya fistula itokanayo na uzazi nchini Kenya. Nimesomewa habari inayotangulia hapo juu na nimekuwa na nafasi ya kuuliza maswali kuhusu utafiti huu. Nimeridhika na jinsi maswali yangu yamejibiwa. Mimi ninatangaza ya kwamba nimeamua kujitolea kwa hiari bila kushurutishwa ili kushiriki katika utafiti huu.

***Mshiriki Utafiti***
 Sahihi:___________________ Au Alama ya Kidole Gumba
Tarehe:__________________
Wakati: _______________________________

***Mtafiti***
Mimi nimemsomea mshiriki uwezo karatasi hii ya habari kwa usahihi, na kwa kadri ya uwezo wangu nimehakikisha kuwa mshiriki anaelewa kuwa yafuatayo yatafanyika:

1. Maelezo yatakayotolewa yatashughulikiwa kwa njia ya siri
2. Utambulisho wake wa kweli hautafichuliwa
3. Uhuru wake wa kujiondoa kutoka utafiti utahakikishwa na hakuna adhabu au karaha itakayo onyeshwa dhidi yake.

Ninathibitisha kuwa mshiriki amepewa nafasi ya kuuliza maswali juu ya utafiti huu na kuwa nimemjibu kwa usahihi. Mshiriki amekubali kushiriki katika utafiti kwa hiari yake na hajashurutishwa vyovyote vile ili kutoa idhini. Mshiriki amepewa nakala ya fomu hii ya ridhaa.

Jina la Mhoji: _________________________Sahihi:______________Tarehe:_______________ Wakati:_________________ Nambari ya kificho ya Mhojiwa ______________________

Nambari ya kificho ya Stesheni ______________

**HEALTH SEEKING BEHAVIOUR AND REINTEGRATION OF PATIENTS WITH OBSTETRIC FISTULA IN KENYA**

**Code No** [___|___|___|___|___] **Site No** [___|___] **Recorder No______________**

**Date: ___________________ Time: Start** [___|___|___|___] **Stop** [___|___|___|___]

**Narrative Guide I: Patients**

**Introduction**

Good morning/ afternoon. My name is ____________. I am carrying out a study on obstetric fistula. The study seeks to investigate the kind of help that women seek while suffering from obstetric fistula and how they settle back into their communities after surgery. As a patient who has undergone surgery for obstetric fistula, I would like to ask you some questions on this topic. Please feel free to answer honestly and provide as much detail as possible to the best of your memory. The conversation will be recorded for transcription later on though I will take some notes during our conversation. Can I proceed?

***Record verbally the Code No and Serial No on the voice recorder.***

1. Tell me about the illness you have had. What were the first symptoms? What did you think when you noticed these symptoms? What caused those symptoms? What did you do?
2. Describe the circumstance around which you developed this condition? *Probe for labour and childbirth experience, skilled birth attendants, TBA*
3. How did you get to know you had this condition?
4. What did you do when you realised you had this condition?
5. Who was the first person in your family did you share information about your illness? What reasons made you share with this person? Are there other people you shared with next and what are the reasons for this?
6. Are you able to recall the people you consulted? Who among these was the first person you sought help from? Who did you seek help from next? What help did they give you? What did you do next? *(Probe from first to last point of person’s participant sought help from).*
7. Describe the help you received from these individual(s)? What help did this individual(s) accord you? What additional help would you have liked to receive?
8. Explain the reasons that led to your choice of the individuals / places you sought help from? *Probe for convenience, beliefs, cultural requirements, authenticity of provider, health and other reasons. Ask this question for all providers mentioned by participant.*
9. During the illness what are the ways that you used to cope with the illness?
10. Where do you routinely/ normally go or whom do you consult when you are ill from other illnesses?

**Dodoso La Maswali ya Wagonjwa Wanougua Fistula**

**Mwongozo Wa Simulizi I (Mgonjwa)**

**Utangulizi**

Habari za asubuhi / mchana. Jina langu ni __________. Mimi ni mwanafunzi katika Chuo Kikuu cha Nairobi na kama sehemu ya masomo yangu mimi ninafanya utafiti juu ya fistula ya uzazi. Utafiti huu unalenga kuchunguza aina ya msaada wanawake hutafuta wakati wakiwa wagonjwa wa fistula itokanayo na uzazi, na jinsi wanavyorudi kukaa katika jamii zao baada ya upasuaji. Tafadhali eleza kwa kina, na kwa kadiri ambavyo unaweza kukumbuka,bila kuwa na uoga wowote. Kama nilivyo kueleza hapo awali, una uhuru wa kutamatisha mahojiano haya wakati wowote tunapoendelea na mazungumzo iwapo utahisi huwezi kuendelea tena. Wakati wa mahojiano, tutatumia kinasa sauti ili tuyachapishe mahojiano haya, hata hivyo, mimi nitakuwa nikiandika kwa ufupi tunapoendelea na mahojiano.***Je tunaweza kuendelea?***

***Maswali***

1. Hebu nieleze kwa kina kuhusu ugonjwa huu/ uliokuwa nao. Je, ni dalli na maumivu gani uliohisi? Je, ulipoona dalili hizi, ulikuwa na mawazo gani? Ulifikiri nini? Ni nini kilichosababisha dalili hizi? Je, ulifanya nini?
2. Elezea mazingira ambayo hali hii ilitokea? (Peleleza ajriba ya uzazi wa mtoto na matukio yaliojiri alipojifungua, mhudumu aliyemzalisha)
3. Elezea jinsi ulivyogundua una hali hii ya fistula?
4. Je, ulifanya nini ulipogundua ya kwamba una hali hii?
5. Ni mtu yupi wa kwanza katika familia yako uliyemweleza kuhusu hali yako? Je, ni kwa sababu gani ulimweleza yeye kwanza? Je, kuna watu wengine uliowaelezakuhusu hali hii katilka familia yako? Je, ni kwa sababu gani uliwaeleza wao?
6. Je, unaweza kumbuka watu au wajuzi ulienda kwao kupata msaada wa matibabu? Hebu nitajie wale uliowatembelea? Ni nani kati yao uliyemtembele wa kwanza? Ni msaada gani aliokupa? Ni nani aiyefuatia? Ulifanya nini baada ya hapo? Elezea ulikoenda na watu ulioshauriana nao ili kupata msaada wa matibabu tangu mwanzo hadi sasa ulipofika hospitalini? *(Peleleza tangu mwanzo hadi muuguzi wa mwisho aliyehudumia mgonjwa huyu)*
7. Mbona wewe ulichagua kwenda kwa mtu huyu wa kwanza uliyeshauriana naye? Je, ni sababu gani uliwatembelea wale wahudumu walio fuatia? *Eleza sababu ambazo zilifanya uwachague wale wahudumu au mahali ulikoenda kutafutatiba kwao?Peleleza kuhusu urahisi wa kupata huduma kwa ajili ya umbali, imani ya mhojiwa, mila ya uponyaji, uhalisi wa mhudumu, sababu za kiafya, na mengineyo. Uliza swali hili kuhusu wahudumu wote wa afya waliotajwa.*
8. Ni msaada gani uliyopewa na watu hawa uliowataja? Je, kwa maoni yako, msaada huu ulitosha?
9. Ni mbinu/mikakati gani ulitumia kukabiliana na hali iliyoletwa na ugonjwa huu?
10. Je, ni wapi unapokwenda kwa kawaida kutafuta matibabu ya magonjwa mengine?

**Serial No___________________**

**Demographic profile: Patients**

*To be filled in after narrative.*

**Code No** [___|___|___|___|___] **Site No** [___|___] **Recorder No______________**

**Date: ___________________ Time: Start** [___|___|___|___] **Stop** [___|___|___|___]

**Introduction**

Good morning/ afternoon. My name is ____________. I am carrying out a study on obstetric fistula. The study seeks to investigate the kind of help that women seek while suffering from obstetric fistula and how they settle back into their communities after surgery. As a patient who has undergone surgery for obstetric fistula, I would like to ask you some questions on this topic. Please feel free to answer honestly and provide as much detail as possible to the best of your memory. The conversation will be recorded for transcription later on though I will take some notes during our conversation. ***Can I proceed?***

**Section I: Demographic/ Social data**

1. How old are you now? *Age in completed year’s (numerals)* [___|___|___]
2. What is the highest level of education you have attained? (*tick one)*

None  primary 1-4  primary 5-8  secondary 1-4

Technical institute  College University

1. How many children do you have: *(numerals)*
2. Alive? _____________________________
3. How many were born before the fistula? __________________
4. How many were born after the fistula? ____________________
5. Died? ______________________________

**Section II: History of Fistula Occurrence**

1. Number of pregnancy when the fistula occurred? ________________ *(record in numerals e.g. 1, 2, 3 etc.)*
2. How old were you when fistula occurred? *(age in years, numerals)*

______________________________________________

1. How long did you live with fistula before treatment? *( numerals)*

*Years_______________ months_______________*

1. Have you had another VVF surgery prior to this one?

Yes  No *(go to Q 10)*

1. If yes, how long ago was the surgery done? *(numerals)*

*Years_______________ months_______________*

1. What was the outcome of the surgery? *(tick one only)*

I was healed completely  I am still incontinent of urine

I still have psychological symptoms

I still experience pain  other (specify) _____________________________

1. In the delivery and labour that caused the current illness, where did you deliver the baby?

Home  TBA  Hospital  other _______________ (specify)

1. If hospital, what is the estimate time in **hours** you spent in labour before reaching hospital? ____________________________________
2. What is the distance in Kilometres from your current home to the nearest health centre or mobile clinic station?

0 -1  2-5  6-10  11-20  21-50 Above 50

1. What was the distance in Kilometres from your home to the nearest health centre or mobile clinic station when the fistula occurred?

0 -1  2-5  6-10  11-20  21-50 Above 50

1. What is the distance in Kilometres from your current home to the nearest district hospital?

0 -1  2-5  6-10  11-20  21-50  Above 50

1. What was the distance in Kilometres from your home to the nearest district hospital when the fistula occurred?

0 -1  2-5  6-10  11-20  21-50  Above 50

1. During your hospital stay which of the following services have you received? (*Tick all mentioned)*

Counselling   Exercises   Physiotherapy

Surgery and clinical care  Nursing care   Health education

Others (specify) i)_______________________________________________________ ii)_______________________________________________________________________ iii)______________________________________________________________________

**Section IV: Locator information *(to be filled in simultaneously with index J****)*

When you get discharged from hospital during your current visit, where is your residential address going to be? County __________________District _____________________ Location _______________ Sub-location __________**­­­______**Village ____________

***To be recorded in Index J only:*** Kindly provide us with your **Telephone contacts** for the researcher to contact you for the follow up interview? **THANK YOU.**

**TABIA YA KUTAFUTA AFYA NA KUWAUNGANISHA NA JAMII WAGONJWA WANAOGUA FISTULA ITOKANAYO NA UZAZI NCHINI KENYA**

**Sehemu ya I: Takwimu za Kidemografia**

**Utangulizi**

Habari za asubuhi / mchana. Jina langu ni __________. Mimi ni mwanafunzi katika Chuo Kikuu cha Nairobi na kama sehemu ya masomo yangu mimi ninafanya utafiti juu ya fistula ya uzazi. Utafiti huu unalenga kuchunguza aina ya msaada wanawake hutafuta wakati wakiwa wagonjwa wa fistula itokanayo na uzazi, na jinsi wanavyorudi kukaa katika jamii zao baada ya upasuaji. Tafadhali eleza kwa kina, na kwa kadiri ambavyo unaweza kukumbuka,bila kuwa na uoga wowote. Kama nilivyo kueleza hapo awali, una uhuru wa kutamatisha mahojiano haya wakati wowote tunapoendelea na mazungumzo iwapo utahisi huwezi kuendelea tena. Wakati wa mahojiano, tutatumia kinasa sauti ili tuyachapishe mahojiano haya, hata hivyo, mimi nitakuwa nikiandika kwa ufupi tunapoendelea na mahojiano.***Je tunaweza kuendelea?***

1. Je, Una umri wa miaka mingapi sasa?
2. Je, ni kipi kiwango cha juu zaidi cha elimu ambacho umehitimu?
    Hakuna  Shule ya msingi 1-4  Shule ya msingi 5-8

Shule ya sekondari  Chuo cha kiufundi  Chuo kikuu.

1. Je, una watoto wangapi:
   walio hai? ____________________________

Waliozaliwa kabla ya fistula? _____________

Wliozaliwa baada ya fistula?________________

waliokufa? ____________________________

**Sehemu ya II: Historia ya Matukio ya Fistula**

1. Je, mimba hii ilikuwa ya ngapi wakati fistula ilitokea? ________________ *(Rekodi katika tarakimu kwa mfano 1, 2, 3 nk)*
2. Je, ulikuwa na umri gani wakati fistula ilitokea?
   ________________________________________
3. Je uliishi kwa muda wa miaka mingapi ukiwa na huu ugonjwa wa fistula kabla ya matibabu?
   Miaka ______________________ Na Miezi _______________
4. Je, umewahi kufanyiwa upasuaji wa ugonjwa wa fistula kabla ya upasuaji huu wa hivi majuzi hapa_____?

Ndio  La (nenda nambari10)

1. Ikiwa ndio, upasuji huo ulifanywa wakati gani kuanzia sasa?

Miaka ___________ Na Miezi _______________

1. Je, matokeo ya upasuaji huo yalikuwa yapi?

Nlipona kabisa Bado nahisi dalili za kutokwa na mkojo au haja kubwa

Bado nina maumivu ya uchungu  Bado nina mawazo mengi kuhusu ugonjwa huu uliyonipata Jingine (Eleza) ____________________

1. Je, katika ile mimba iliyosababisha fistula uliyotibiwa kwayo sasa, wewe ulijifungulia wapi yule mtoto?

Nyumbani Mkunga wa kienyeji  Hospitalini Nyingine

1. Ikiwa ni hospitalini, je hospitali hii ipo umbali wa kadri **masaa** mangapi unaposafiri? _________________________
2. Je, unaishi umbali wa Kilomita ngapi kutoka kituo cha afya kilicho karibu / kituo cha kliniki ya kuhama?
    0 -1  2-5  6-10 11-20  21-50
3. Je, wakati ulipata huu ugonjwa wa fistula ya uzazi, ulikuwa unaishi umbali wa Kilomita ngapi kutoka kituo cha afya kilicho karibu / kituo cha kliniki ya kuhama?
    0 -1  2-5  6-10 11-20  21-50
4. Je, unaishi umbali wa Kilomita ngapi kutoka hospitali ya wilaya?

0 -1  2-5  6-10 11-20  21-50

1. Je, wakati ulipata huu ugonjwa wa fistula ya uzazi, ulikuwa unaishi umbali wa Kilomita ngapi kutoka hospitali ya wilaya?

0 -1  2-5  6-10 11-20  21-50

1. Wakati umekuwa hospitalini humu, ni huduma gani ambazo umepokea kati ya zifuatazo? *(Weka alama ya vema kwa zote zilizotumika)*

Ushauri nasaha  Mazoezi  viungo vya mwili  upasuaji na huduma ya kitabibu  Huduma ya uguzi  huduma ya elimu ya afya

Nyinginezo *(taja)*

i) _________________________________________________________________

ii) ____________________________________________________________________ iii) ________________________________________________________________

**Sehemu ya III: Maelezo kwa ajili ya kumpata mshiriki**

Tafadhali eleza anuani yako ya makazi unayoelekea baada ya kuruhusiwa kwenda nyumbani

Kaunti ya______________________ Wilaya ya _________________________________ Lokesheni _____________________ Lokesheni ndogo _________________________ Kijiji ______________________

**Sehemu hii ijazwe mtawalia na Index J:** Tafadhali niarifu **nambari ya simu** yako kwa ajili ya mtafiti kuwasiliana nawe wakati wa mahojiano ya pili ukiwa nyumbani?

**ASANTE**
